# Application of Wireless Intelligent Control System for HPS Lamps and LEDs Combined Illumination in Road Tunnel

**DOI:** 10.1155/2014/429657

**Published:** 2014-12-21

**Authors:** Jinxing Lai, Junling Qiu, Jianxun Chen, Yaqiong Wang, Haobo Fan

**Affiliations:** ^1^Shaanxi Provincial Major Laboratory for Highway Bridge & Tunnel, Chang'an University, Xi'an 710064, China; ^2^School of Highway, Chang'an University, Xi'an 710064, China; ^3^School of Electronics and Control Engineering, Chang'an University, Xi'an 710064, China

## Abstract

Because of the particularity of the environment in the tunnel, the rational tunnel illumination system should be developed, so as to optimize the tunnel environment. Considering the high cost of traditional tunnel illumination system with high-pressure sodium (HPS) lamps as well as the effect of a single light source on tunnel entrance, the energy-saving illumination system with HPS lamps and LEDs combined illumination in road tunnel, which could make full use of these two kinds of lamps, was proposed. The wireless intelligent control system based on HPS lamps and LEDs combined illumination and microcontrol unit (MCU) Si1000 wireless communication technology was designed. And the remote monitoring, wireless communication, and PWM dimming module of this system were designed emphatically. Intensity detector and vehicle flow detector can be configured in wireless intelligent control system, which gather the information to the master control unit, and then the information is sent to the monitoring center through the Ethernet. The control strategies are got by the monitoring center according to the calculated results, and the control unit wirelessly sends parameters to lamps, which adjust the luminance of each segment of the tunnel and realize the wireless intelligent control of combined illumination in road tunnel.

## 1. Introduction

**Figure 1:**
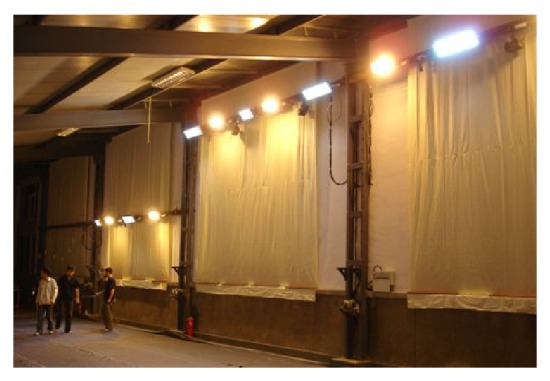
Illumination experimental laboratory [7].

**Figure 2:**
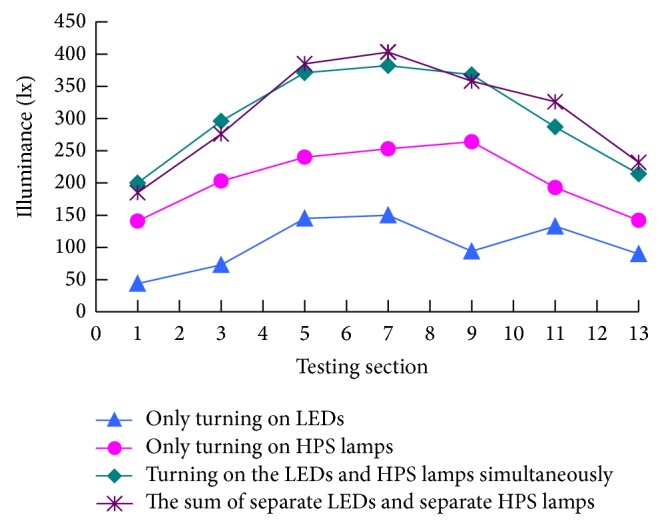
Illuminance of light source versus testing section.

As we know, the road tunnel, with closed top and sides, is a special kind of tubular structure. The environment in the tunnel, compared with the outside environment, has the following characteristics: high brightness contrasts, poor air quality, low visibility, and so forth. Due to the particularity of the environment in the tunnel, the reasonable tunnel illumination system should be developed, which can give drivers a comfortable visual environment in the tunnel [1]. Differentiated from railway tunnels and underwater tunnels, the driving conditions of road tunnels are more easily affected by tunnel illumination. The tunnel illumination must consider the human eye's dark adaptation and light adjusting so as to ensure the safety of driving traffic into the tunnel. And on this basis, in order to eliminate the “black-hole effect” and “white-hole effect” at entrance and exit of highway tunnel [2], some improvement measures have been effectively implemented by means of improving the tunnel illumination system, which are to establish a relatively stable and moderate decreasing CIE adaptation curve [3] as far as possible between the luminance of electric-light source in the tunnel and the outside luminance of natural light. At present, HPS lamps are widely used in the majority of the road tunnel illumination. Conventional HPS lamps have a long working life, high luminous efficacy, high color temperature, and more comfortable environment [4]; however, the actual service life will shorten sharply when the luminous output changes unsteadily as the voltage fluctuates; in addition, the conventional operation of highway tunnel is restricted by the large electric energy loss, frequent replacement of lamps, light pollution, and thermal radiation produced by the intense light. As an upgrading light source in the 21st century, LEDs, comparing the HPS lamps or other existing light sources, provide a much longer lifetime (more than 5 times longer than HPS lamps), high luminous efficiency, and more adjustable, more controllable, and efficient form of lighting [5]. What is more, LED solutions offer energy savings of as much as 50%, which are becoming more and more popular. LEDs, however, have two significant challenges in high price and heat dissipation in tunnel illumination system. With high efficiency and good color rendering [6], LED solutions can shorten the adaptation time when driving into the tunnel in the daytime. For the considered efficiency, 90 percent of the power used by an LED is converted into light. The combined illumination system [7], which has both advantages of HPS lamps and LEDs, is proposed. And the operation cost of the tunnel can be effectively reduced. The combined illumination system meets the requirements of tunnel illumination by means of adjusting the distance between the different lamps and reasonable arrangement of light distribution in the tunnel entrance and transition segment [8]. The interrelation of the illuminance of the light sources with different color temperature and color rendering index is the principal problem to be resolved for tunnel combined illumination system. Therefore, the tunnel illumination lab, as shown in Figure 1, has been established by our group [7], which is used to explore the interaction between different light sources. Consequently, in the lab we have demonstrated that the test result with separate LEDs and separate HPS lamps is on the same level with the test value when LEDs and HPS lamps are concurrently turned on, and the testing results are shown in Figure 2. The relationship between the two, without considering the impact of the measuring error, can be considered as the linear additivity property.

Over the last decades, with the rapid development of industrial automation and the operation management of highway tunnel, the illumination control technology in highway tunnel has been continuously in progress. Highway tunnel illumination control mode has gone through two stages successively, which are manual control mode and automatic control mode [9]. Currently, a new period of the tunnel illumination control system is approaching, which is developing in the direction of integration, networking, and intelligence. What is the intelligent control mode? Intelligent control technology, consisting of artificial intelligence, expert system, fuzzy control algorithm, neural network, genetic algorithm, and so forth, is applied in this intelligent control system. According to the highway tunnel CIE adaptation curve, the intelligent control mode can dynamically adjust the luminance to be safe, comfortable, efficient, and economical. More specifically, the lighting adjusted according to the requirement (with the standard requirement) can come true [10]. For this purpose, the wireless intelligent control system based on HPS lamps and LEDs combined illumination and microcontrol unit (MCU) Si1000 wireless communication technology is proposed.

## 2. Wireless Intelligent Control System

### 2.1. System Architecture

**Figure 3:**
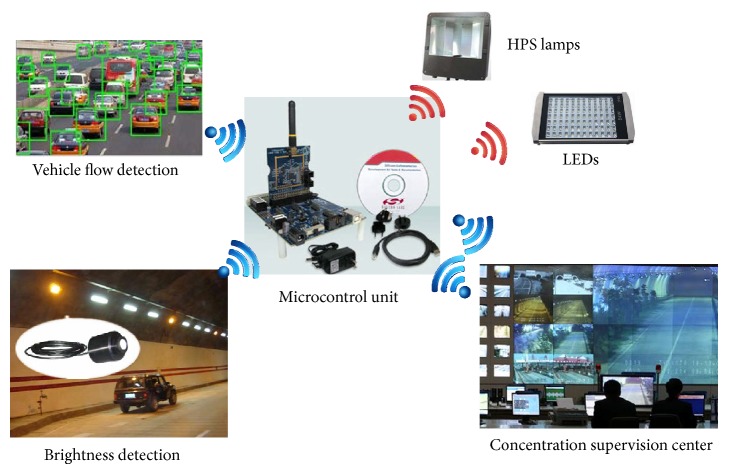
The tunnel illumination intelligent control system network structure.

This system is mainly composed of five parts [11], which are tunnel illumination network, brightness detection, vehicle flow detection, field surveillance unit (FSU), and concentration supervision center (CSC), as shown in Figure 3. And the tunnel illumination network is composed of HPS lamps and LEDs, which is controlled through wireless protocols; furthermore, it is the major component of the tunnel illumination intelligent control system. The brightness detection is designed to detect the luminance and illuminance in tunnel and transmit in real time the monitoring data to FSU. Therefore, the luminance in tunnel can be regulated by FSU. The vehicle flow detection is designed to detect the vehicle flow in real time, also, transmit the monitoring data to FSU, and then adjust the brightness of the corresponding lamp in tunnel by FSU. Meanwhile, CSC is designed to collect the parameters of luminance and illuminance, lamps working conditions, and vehicle flow in tunnel. The entire illumination system is monitored, which has two major components: monitoring computers and monitoring software. FSU, adjusting control of lamps in tunnel on the basis of the parameters set by superior master unit, transmits in real time the executive monitoring commands and data to host computer by wireless signal technology.

### 2.2. Working Principle of the System

The working principle of wireless intelligent control system for HPS lamps and LEDs combined illumination in road tunnel is that the luminous intensity of illumination crucial points in tunnel is detected by intensity detectors installed inside and outside the tunnel. As well as the vehicle flow, vehicle speed and other information are detected by vehicle flow detectors installed in the tunnel illumination transition segment. The regulation calculation system, which gathers this information, put forward the corresponding control strategy. The illumination situation of all segments in tunnel is regulated constantly through the adjustment of luminous intensity of lamps. Smooth transition illumination makes the eye adapt to the change of luminous intensity as soon as possible, which eliminates the influence of bright and dark gap on the visual blind area [12]; what is more, the efficiency of illumination and energy saving are improved. First of all, the initialization and calibration need to be set after the system is installed. At this stage, the real-time information, consisting of tunnel access zone luminance [13], vehicle speed, vehicle flow, and so on, will be stored in the data storage unit, which is obtained through tunnel monitoring equipment. Meanwhile, the relational data, including outside illumination detectors, the cave illumination detectors, artificial illuminance detectors, and driving current of lamps, will be also saved in the data storage unit in the process of calibration. When the system enters the working state, the monitor data of tunnel access zone luminance are queried by the master controller system. At the same time, the information of vehicle speed and vehicle flow in traffic video detection instrument is read through field-bus according to the preset detection interval. Then, the preset data are calculated and queried by the master controller, and corresponding control strategies are put forward according to the detection data. After that, the data transmitting equipment transmits control-trend signal to the wireless tunnel light web step by step. Each tunnel lamp is identified according to the addressing scheme. When the address is confirmed, the controller will correct the constant-current supply parameters, which can adjust the brightness of LEDs. Then, the monitor data of luminance in the tunnel are queried by the master controller system using the field-bus, which are compared with the target luminance and regulated constantly [14]. When the difference range reaches the default value, regulation will be terminated; otherwise, the control-trend signal will continue to be transmitted to the power receiving unit.

## 3. Design of TT&C Hardware System

**Figure 4:**
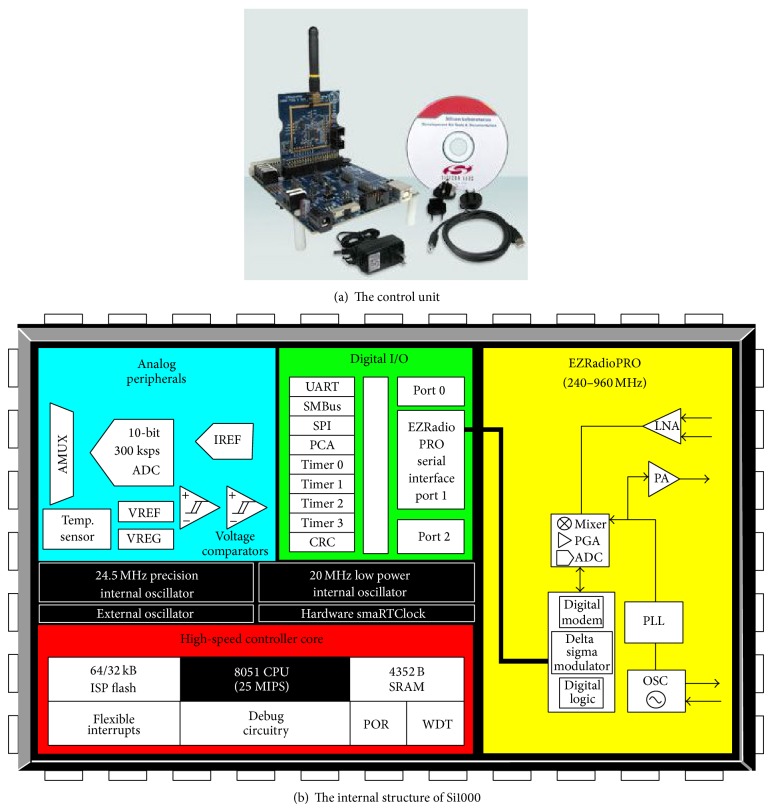
MCU Si1000 control unit.

The single-lamp controller in wireless constant-current dimming control system is the core part of the TT&C system. Sil000 device is a fully integrated mixed-signal system-on-a-chip MCU, as shown in Figure 4, which is the control chip in master control block. In addition, it integrates ultra-low-power C805IF930 MCU and high transmittance Si44320 RF chip, which is marketed by* Silicon Labs Corp*. [15]. And the controller is composed of four modules, the power management module, the signal acquisition module, the RF wireless module, and the constant-current driver.

The signal acquisition module is mainly composed of two parts, voltage acquisition and current acquisition. Voltage signals and current signals in circuit are acquired so as to monitor and store the state of each lamp (on, off, or fault) and calculate the parameters of equipment. Wireless Sil000 MCU is the main part of RF wireless module, and the primary functions of it include AD conversion, PWM output, data communication, and the controller switches. Specifically, the data communication can be also divided into two parts, which are receiving commands of host computers and uploading own data. When receiving the commands of host computers, PWM output and data communication will, respectively, execute the PWM dimming commands and on-off control. Moreover, the existing module is used by constant-current driver, which can select the corresponding constant-current drivers according to the needs of lamps.

**Figure 5:**
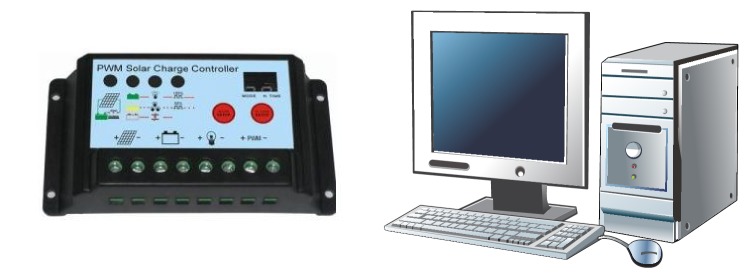
The PWM dimming control module.

**Figure 6:**
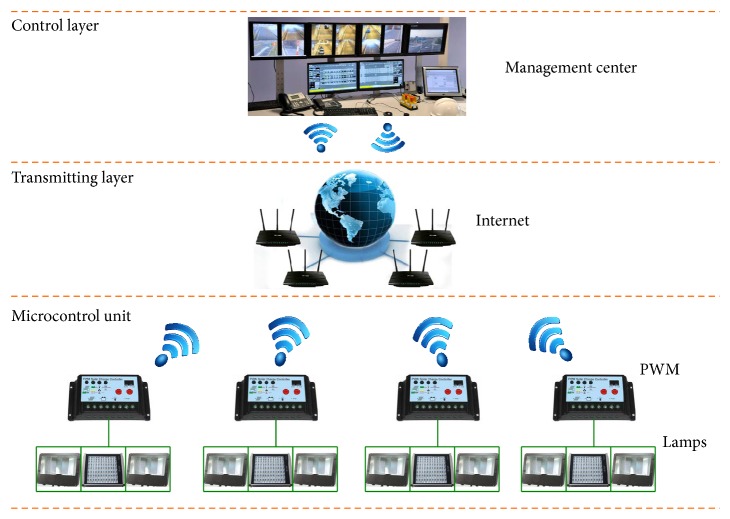
The dimming control system structure based PWM.

The wireless constant-current dimming control system [16] can be mainly classified into five modules according to its functions, which are serial port communication module, signal acquisition module, wireless communication module, PWM dimming control module (Figure 5), and on-off control module. Specifically, the wireless communication module consists of the wireless data sending and receiving. The two-layer network control framework is adopted in the dimming control system. Many dimming controllers are installed along the tunnel axis, and the controllers are interconnected by fiber optic redundant Ethernet, which are connected to the illumination control workstation in management center. The dimming controller with four channel RS485 buses, respectively, connected to the basic lamps in the region, can achieve the dynamic dimming control and inspection for lamps in the tunnel. Meanwhile, it can avoid the zebra effect owing to the operation of turning off the light unilaterally [17]. Master-slave protocol is used by the field-bus communication equipment and the dimming controller is the master station, while the lamp is the slave station. In addition, this system can be connected to the comprehensive monitoring system by reserving interfaces. The dimming control system structure is shown in Figure 6.

## 4. Design of System Software

**Figure 7:**
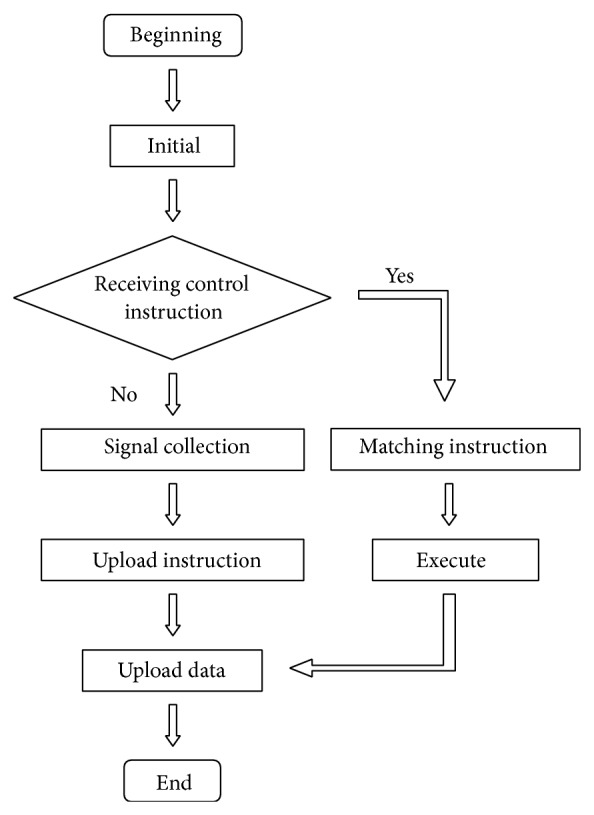
Main program flow chart.

Taking into account that the central controller fails, this will directly lead to all tunnel lamps being out of control. The modularized structure is used in the design of software system. KEIL C51 is developed through 51 series MCU produced by KEIL Software Company. The development schemes based on various functions and emulator-based debuggers are combined together by using KEIL C Uvision integrated developing environment, which provide a powerful integrated development debugging tool and abundant resources. The Uvision is a new powerful integrated developing environment (IDE) for Windows, which can finish the whole development process of editing, compiling, connecting, debugging, and simulation. Firstly, IDE itself or other editors are used to edit the C programming and compile the assembler. Then, the standard HEX files are generated, which can process the debugging of source codes. Finally, the debugged source codes are written in chip-FLASH through simulator. The main program flow is shown in Figure 7.

## 5. Application Effect Evaluations

During the actual road tunnel operation period, the luminance outside the tunnel changes unsteadily with the variation of weather, season, and time, which induces a large electric energy loss in tunnel. However, the wireless intelligent control system based on combined illumination and microcontrol unit (MCU) Si1000 wireless communication technology can dynamically adjust the luminance to be safe, comfortable, efficient, and economical.

**Figure 8:**
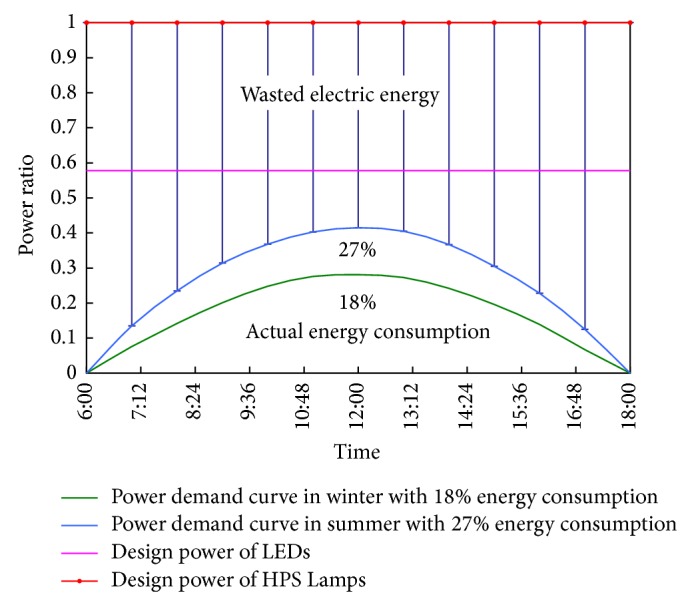
Relative ratio curves of the light-adjusting power and energy consumption in the lighting strengthened section.

According to the actual application effect of this intelligent control system in a tunnel, Yunnan province, China, the luminance is softness in the tunnel entrance segment with the interaction between HPS lamps (white light) and LEDs (yellow light), and the visibility at the tunnel exit as well as the pavement illuminance in the tunnel has met the design requirements. Additionally, taking the operation cost of the tunnel into consideration, it can be seen that this intelligent control system can cut down unnecessary expenses to a large extent. The relative ratio curves of the light-adjusting power and energy consumption are shown in Figure 8, which presents energy-efficient benefits of the tunnel intelligent illumination control system. The area above the curve means the wasted electric energy, which is nearly 3 times of the actual energy consumption. Even the energy consumption of LEDs with four-level light-adjusting pattern is approximately 2 times of the actual energy consumption. Thus, employing this intelligent illumination control system, the lighting adjusted according to the requirement (with the standard requirement) can come true.

## 6. Conclusions

In the paper, the wireless intelligent control system for HPS lamps and LEDs combined illumination in road tunnel is proposed to solve the problem of high illumination costs and high energy consumption. The HPS lamps and LEDs combined illumination system is utilized in the lighting strengthened section, which can reduce the effect of the cold and hot light illuminator on tunnel entrance section and meet the requirements of tunnel illumination. Meanwhile, the LEDs installed in the tunnel basic segment can effectively reduce the operation cost.

The wireless intelligent control system has been successfully implemented for the first time in China, which can achieve the purpose of energy saving and the purpose of intelligent control. The intelligent control system, adopting the remote monitoring technology, wireless communication technology, and PWM dimming module, can easily finish the information and command transmission, which is mainly composed of tunnel illumination network, brightness detection, vehicle flow detection, field surveillance unit (FSU), and concentration supervision center (CSC). The construction intensity and construction cost are curtailed, and, additionally, the maintenance of the illumination system is convenient. On the other hand, together with the application of wireless transmission technology, no signal lines exist between the lamps, the flexibility and extensibility of the system are relatively great, and the control method and structure configuration are extremely flexible.

In summary, assuming the present dynamics of the development of intelligent control technology, some further study on the application of wireless intelligent control system for HPS lamps and LEDs combined illumination in road tunnel is carrying on, and the function mechanism of this intelligent control system presented for this study can serve as reference for high efficiency design and energy saving of the tunnel illumination system.

## References

[1] Wang S. F., Deng X., Wu X. L. (2010). Overview of lighting control techniques in expressway tunnels. *Technology of Highway and Transport*.

[2] Professional Standards Compilation Group of People's Republic of China (1999). *Specifications for Design of Ventilation and Lighting of Highway Tunnel (JTJ 026.1-1999)*.

[3] International Commission on Illumination (CIE) (2004). *Technical Report 188-2004. Guide for the Lighting of Road Tunnels and Underpasses*.

[4] Wencheng C., Zheng H., Liping G., Yandan L., Dahua C. (2008). Performance of induction lamps and HPS lamps in road tunnel lighting. *Tunnelling and Underground Space Technology*.

[5] Eigentier K. (2006). Experiences with LED-based visual guidance systems in tunnels. *Tunnelling and Underground Space Technology*.

[6] Yager R. R. (1988). On ordered weighted averaging aggregation operators in multicriteria decision making. *IEEE Transactions on Systems, Man, and Cybernetics*.

[7] Wang Y. Q., Lai J. X., Xie Y. L. (2009). Experimental research and application of high voltage sodium lamp and low emitting diode combined lighting in tunnel. *Chinese Journal of Underground Space and Engineering*.

[8] Liu L. S., Lin W., Wang P. Z. (2010). The application and analysis of LEDs for the urban tunnel lighting. *China Illuminating Engineering Journal*.

[9] Xu J. F. (2010). Opportunities and challenges of LED lights source in the tunnel lighting. *Lamps and Lighting*.

[10] Xi F. (2011). Application of LED lighting lamps in highway tunnels. *Highway*.

[11] Wang Y. Q. (2007). *Research Report of Lighting and Security Technology Research in Double Traffic Tunnel*.

[12] van Bommel W. (1981). Tunnel lighting practice world-wide. *Lighting Research & Technology*.

[13] Adrian W. (1982). Investigations on the required luminance in tunnel entrances. *Lighting Research and Technology*.

[14] Zeng H., Qiu J., Shen X., Dai G., Liu P., Le S. (2011). Fuzzy control of led tunnel lighting and energy conservation. *Tsinghua Science and Technology*.

[15] Silicon Labs http://www.silabs.com/products/wireless/wirelessmcu/Pages/Si1000.aspx.

[16] Burgio A., Menniti D. (2013). A novel technique for energy savings by dimming high pressure sodium lamps mounted with magnetic ballasts using a centralized system. *Electric Power Systems Research*.

[17] Narisada K. (1975). Applied research on tunnel entrance lighting in Japan. *Lighting Research and Technology*.

